# Carbon Stocks and Fluxes in Tropical Lowland Dipterocarp Rainforests in Sabah, Malaysian Borneo

**DOI:** 10.1371/journal.pone.0029642

**Published:** 2012-01-03

**Authors:** Philippe Saner, Yen Yee Loh, Robert C. Ong, Andy Hector

**Affiliations:** 1 Institute of Evolutionary Biology and Environmental Studies, University of Zurich, Zurich, Switzerland; 2 School of International Tropical Forestry, Universiti Malaysia Sabah, Sabah, Malaysia; 3 Forest Research Center Institute, Sabah, Malaysia; Centre National de la Recherche Scientifique, France

## Abstract

Deforestation in the tropics is an important source of carbon C release to the atmosphere. To provide a sound scientific base for efforts taken to reduce emissions from deforestation and degradation (REDD+) good estimates of C stocks and fluxes are important. We present components of the C balance for selectively logged lowland tropical dipterocarp rainforest in the Malua Forest Reserve of Sabah, Malaysian Borneo. Total organic C in this area was 167.9 Mg C ha^−1^±3.8 (SD), including: Total aboveground (TAGC: 55%; 91.9 Mg C ha^−1^±2.9 SEM) and belowground carbon in trees (TBGC: 10%; 16.5 Mg C ha^−1^±0.5 SEM), deadwood (8%; 13.2 Mg C ha^−1^±3.5 SEM) and soil organic matter (SOM: 24%; 39.6 Mg C ha^−1^±0.9 SEM), understory vegetation (3%; 5.1 Mg C ha^−1^±1.7 SEM), standing litter (<1%; 0.7 Mg C ha^−1^±0.1 SEM) and fine root biomass (<1%; 0.9 Mg C ha^−1^±0.1 SEM). Fluxes included litterfall, a proxy for leaf net primary productivity (4.9 Mg C ha^−1^ yr^−1^±0.1 SEM), and soil respiration, a measure for heterotrophic ecosystem respiration (28.6 Mg C ha^−1^ yr^−1^±1.2 SEM). The missing estimates necessary to close the C balance are wood net primary productivity and autotrophic respiration.

Twenty-two years after logging TAGC stocks were 28% lower compared to unlogged forest (128 Mg C ha^−1^±13.4 SEM); a combined weighted average mean reduction due to selective logging of −57.8 Mg C ha^−1^ (with 95% CI −75.5 to −40.2). Based on the findings we conclude that selective logging decreased the dipterocarp stock by 55–66%. Silvicultural treatments may have the potential to accelerate the recovery of dipterocarp C stocks to pre-logging levels.

## Introduction

The lowland rain forests on the island of Borneo are recognized as a living carbon density hotspot [1], with an average aboveground biomass that is roughly 60% (457.1 Mg ha^−1^) higher than the Amazonian average of 288.6 Mg ha^−1^ [2]. Most of Borneo was covered with tropical evergreen rainforest until the 1950s [3] but an approximate annual deforestation rate of 1.7% has decreased total forest cover to 57% of the original area by 2002 [4]. The subsequent carbon C loss associated to deforestation is a representative trend for the whole Indo-Malaya region, including South Asia, Southeast Asia and Papua New Guinea, were forest cover was less than 40% of the original area by 2000 [5]. Consequently, ongoing exploitation for timber is an important source of C emissions and efforts are proposed to reduce C release to the atmosphere from deforestation and degradation (REDD+) [6]. However, there is still considerable uncertainty about C stocks [7] and fluxes [8] and their subsequent losses and accumulation rates in tropical rain forests, in particular for South East Asia [9]. An adequate understanding of the state of the remaining mixed dipterocarp forests and present C stocks and flows at the regional and local level is therefore needed [10].

**Figure 1:**
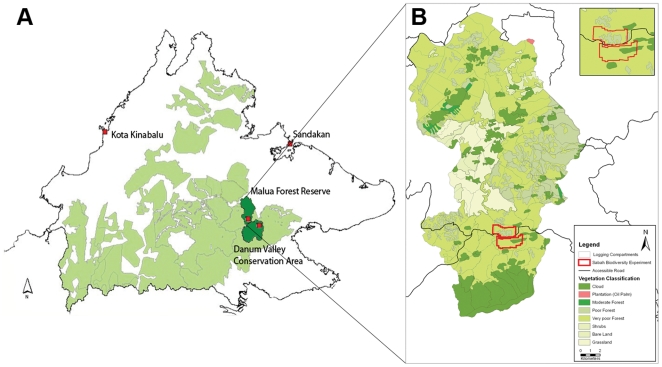
Map of Sabah and forest quality map of Malua Forest Reserve. (A) Allocated production forest is shown across Sabah (light green, also including Malua Forest Reserve). The study sites (marked in red) are located in the Malua Forest Reserve and the Danum Valley Conservation Area (dark green). (B) Forest quality map of the Malua Forest Reserve (visual interpretation of aerial photographs, 1∶17,500). The boundary of the Sabah Biodiversity Experiment (500 ha) is outlined (red). Forest classification is based on number of trees ≥60 cm DBH derived from crown size. Cloud forest: >16 trees ha^−1^, Moderate: 9–16 trees ha^−1^, Poor: 5–8 trees ha^−1^, Very Poor: 1–4 trees ha^−1^, Shrubs/Bare Land/Grassland: none, Plantation: Oil palm monoculture.

An overview of recent forest carbon stocks in tropical regions is given elsewhere, where they reported a total aboveground C (TAGC) estimate of 164–196 Mg C ha^−1^ for Malaysia [7]. For the Malaysian state of Sabah (North Borneo), where this study was undertaken, logging played an important role in the tiger economy, particularly in the 1980s [11]. After decades of commercial timber exploitation and subsequent conversion of the land cover to palm oil plantations (*Elaeis guinensis*) 53% of the original forest coverage of Sabah remains [12] with less than 15% in an undisturbed primary forest state. The secondary production forests are in various stages of post-logging condition, ranging from almost pristine to selectively logged and even completely degraded (Fig. 1).

In this paper, we estimate impacts of logging on components of the C balance of an area of secondary production forest of the Malua Forest Reserve that is the location of the Sabah Biodiversity Experiment. We present it in the context of previous work made in the neighbouring primary lowland rainforest of the Danum Valley Conservation Area as well as to other forests in the region. Here we ask how selective logging has impacted C stocks and fluxes after 22 years and predict C losses for the area of study. This can later be used as a baseline to study changes in the C balance due to enrichment planting with dipterocarp seedlings and other management techniques.

## Materials and Methods

### Location

The selectively logged and unlogged forest study sites are located within lowland dipterocarp forest of East Sabah, Malaysian North Borneo (Royal Society South-East Asia Rainforest Research Programme Project No. RS243) (Fig. 1A). The region is aseasonal with an annual rainfall of >3000 mm [13]. The forest covers a concession of one million hectares which belongs to the publicly owned Sabah Foundation. Most of the area has been selectively logged twice, once in the 1980s and once within the last ten years. An area of unlogged primary forest–the Danum Valley Conservation Area–was left at the heart of the concession. Our unlogged forest site is in this area, along the main trail of the grid system to the west (W6–W9) and less than 1 km from the Danum Valley Field Centre (N05°19′21″ E117°26′26″, 220–284 m.a.s.l). A general description for the soil, geology and vegetation of Danum Valley is given elsewhere [14]. The logged forest site is part of the Sabah Biodiversity Experiment (N05°05′20″ E117°38′32″, 102 m.a.s.l.) which is located in the southern part of the Malua Forest Reserve, a 35,000 ha area of selectively logged production forest that lies immediately to the North of Danum Valley (22.6 km air distance from the Danum Valley Field Centre) (Fig. 1B). The Malua Forest Reserve was selectively logged during the early 1980s (and relogged in 2007, with the exception of the area used for this study that was established in 2003 and therefore excluded from the second round). Yearly production volume, based on data from logging coupes of the same region (Lahad Datu district), were reported as 95–157 m^3^ ha^−1^ (1970–1980) and 75–134 m^3^ ha^−1^ (1980–1990) (unpublished data, Danum Valley Field Centre). Our estimates of logged forest are for the background vegetation of the Sabah Biodiversity Experiment, a forest restoration project, that covers 500 ha forest that was selectively logged in 1986. The large-scale experiment examines impacts of dipterocarp diversity on forest structure and functioning [15]–[18].

Site characteristics, including topography, soil characteristics and estimated pre-logging timber volume are comparable at both sites (selectively logged and unlogged) before logging (Table 1 and Table S1). Hence, we assume that changes in basal area and C stocks in the two different forest types largely indicate the effect of selective logging, rather than site specific differences in pre-logging conditions.

**Table 1:** Site characteristics of a pre-logging survey completed in 1983.

	Danum	Malua
**Elevation**	<250 m	<250 m
**Topography**	Slopes 15–25°	Slopes 0–20°
**Parent Material**	Mudstone	Mudstone
**Soil Type**	Orthic acrisol	Orthic acrisol
**Soil Family**	Tanjong Lipat	Tanjong Lipat
**Soil Association**	Bang	Kretam and Mentapok
**Est. Pre-logging Volume**	178–230 m^3^ ha^−1^	193–221 m^3^ ha^−1^
**Est. Dipterocarp Volume**	149–225 m^3^ ha^−1^	180–216 m^3^ ha^−1^

Site descriptions for both forest types (selectively logged and unlogged) were extracted from Yayasan Sabah Forest Management Plan 1984–2032 (unpublished data).

### Components of the C balance of the logged and unlogged forests

We estimate the main C stocks, the minor C stocks and the measured C fluxes, missing to close the C balance are wood net primary productivity (NPP) and autotrophic respiration. The components were measured on site or estimated from existing biomass regression models and biomass partitioning ratios using methods given below. A less extensive survey was made for the unlogged primary forest of Danum Valley using identical regression models to estimate total aboveground tree biomass. Stocks and fluxes measured in selectively logged forest were compared to existing published literature from the unlogged primary forest at Danum Valley.

### Aboveground tree basal diameter

The tree inventory was taken in July 2008 in the selectively logged forest of the Sabah Biodiversity Experiment (areas excluded from enrichment planting were used for the inventory) and in unlogged forest close to the Danum Valley Field Centre (Fig. 1). The size of the sampled areas in both forest types was one hectare divided into 4 replicate transect lines, each 250×10 m (100 m apart). Lines and orientation (North-South) were measured with a handheld GPS to 10 m accuracy (GPSMAP 60 CSx, Garmin, USA). All trees >10 cm diameter breast height (DBH at 130 cm) that were within five meters on each side of the transect line were tagged, measured with a DBH tape (Yamayo, Japan) and identified to species or, if unknown, to genus level (Table S2). We followed the RAINFOR field manual [19] to measure all trees in a comparable standardized way. If a tree showed large buttresses its DBH was measured just above using a ladder.

### Wood density estimation

Species specific wood density estimates from the World Agroforestry Centre Wood Density Database (WAC; Table S2) were used and cross referenced with on site measurements on a subset of 18 local tree species (n = 32, R^2^ = 0.81; unpublished data). For our own measurements wood density was defined as the oven-dry weight of wood divided by its wet volume [20]. Wood cores were taken with an increment borer at 130 cm height (Haglöf, Sweden) on trees that were located outside of the survey lines. Wood density values were calculated using the water-displacement method described in [21]. If the species was unknown or the wood density not available we took mean wood density of the genus as a substitute. In the few cases (<4 cases) where genus wood density was not available a mean overall wood density of 0.6 g cm^−3^ was taken.

### Total aboveground tree biomass and C stocks

An established DBH-AGB regression model was used to estimate tree biomass and C stocks in logged and unlogged forest [22]. The general form of the equation is:
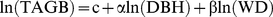
(1)where TAGB is in kg tree^−1^, DBH is in cm, c (−0.744) is the intercept, α (2.188) and β are the slope coefficient of the regression for mixed species forest and WD is wood density in g cm^−3^.

Several other published allometric models were considered for direct comparison [23]–[26]. As site specificity is known to be of importance for the selection of allometric models [22], all else being equal we took the DBH-AGB regression model from research on the geographically closest study area. This choice led to lowest estimation for aboveground tree biomass and is therefore probably a conservative approach. All reported standard errors describe the variation among the 4 transect lines in each forest type (logged, unlogged) and do not incorporate error in allometric equations. Carbon content was assumed to be 50% by total biomass for trees and understory vegetation [24].

### Total belowground tree biomass and C stocks

Coarse root biomass (>2 mm diameter) was estimated based on values derived from Pasoh Forest Reserve (peninsular Malaysia), where they reported a BGB/AGB biomass partitioning ratio of 0.18 [27]. Carbon content was assumed to be 50% by biomass [28].

### Standing deadwood

DBH, height, degradation state (not degraded, degraded, heavily degraded) and tree structure (stem only/stem plus branches) was noted for all dead standing trees in the selectively logged forest that were within five meter on each side of the transect lines. The height of dead standing trees was estimated visually and tree volume was calculated following the methods described in [29], [30]. Due to the lack of correct wood density estimates for sampled dead trees a wood density of 0.5 g cm^−3^ was assumed as reported from Venezuela [31].

### Soil organic matter

Soil organic C from plant material was estimated from randomly located sites and represented large scale spatial variability. Soil core samples were taken from the middle of 13 unplanted 4 ha control plots in the selectively logged forest of the Sabah Biodiversity Experiment in June 2006. At each plot three 1 m soil pits were excavated, each located 10 m away from the middle of the plot directed either South, Northwest or Northeast. Soil cores (100 cm^3^) were inserted horizontally down to 1 m depth and sub-samples taken from 11 different layers (0–5, 5–10, 10–20, 20–30, 30–40, 40–50, 50–60, 60–70, 70–80, 80–90, 90–100 cm) using standard soil corers (Eijkelkamp, Netherlands) and a rubber hammer (n = 396). The three replications (South, Northwest and Northeast) of each layer in each plot were pooled for subsequent soil analysis to include site variability. Damp soil samples were laid out to dry in trays in a well ventilated room until the soil sample weight became quite consistent (about 5 days to a week depending on the initial conditions of the samples). Rock and root components were separated from each sample. Weight and volume was determined using the water displacement method for rocks and using ethanol displacement, with a lower density for estimating fine root volume. Bulk density was derived from the equation:
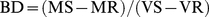
(2)where BD is bulk density (g cm^−3^), MS is mass of the dry soil (g), MR is mass of rocks and roots (g), VS is volume of dry soil (ml) and VR is volume of rocks and roots (ml). Samples were further ground with a porcelain mortar and pestle to pass through a 2 mm sieve. C content was determined by the Walkley-Black method, a wet chemical analysis [32], [33].

### Understory vegetation

Six quadrats (5×5 m) along each of 4 transect lines in the selectively logged forest were randomly selected (n = 24). Within the quadrat saplings (<10 cm DBH and >2 m height), seedlings (<2 m height) and all woody vines were harvested. All saplings were individually measured using a common spring scale. Wet biomass for seedlings was measured once for all seedlings together. A subsample of saplings, seedlings and woody vines was dried to constant mass (7 days, 60°C) to relate wet to dry biomass (y = 0.55x, R^2^ = 0.98, n = 76, intercept was set to zero). One *Eusideroxylon zwageri* (belian, IUCN status: vulnerable) sapling was not harvested due to its age and size (probably about 30 years, 5.2 cm DBH).

### Standing litter

Within each quadrat 0.5×0.5 m subquadrats were established to collect all woody debris and leaf litter separately. The low estimate of this study may indicate that the method of collecting all woody debris within 24 subquadrats (0.5×0.5 m^2^) did not capture large downed woody debris adequately and that other proposed methods should be considered [30], [34].

### Fine root biomass

In each subquadrat of 0.5×0.5 m vertical soil cores (100 cm^3^) at the soil surface were taken (0–5 cm) using standard soil corers (Eijkelkamp, Netherlands). Fine roots (≤2 mm diameter) were extracted by washing the soil cores over a 210 µm sieve (Retsch, Germany). All collected samples were dried in a glasshouse (7 days, 60°C) before measuring their dry biomass with a precision scale.

### Litterfall

Litterfall traps (1 m^2^) were randomly allocated along the 4 transect lines in the selectively logged forest at 130 cm height, using fine meshed plastic net (n = 40). The sampling complies with previously proposed standards, including a minimal sampling duration of 1 year, a total sampled surface of 40 m^2^ and a trap surface of more than 0.25 m^2^ [35], [36]. Litter was collected every other week over one year (June 2006–June 2007, n = 25) and was further separated into leaves, twigs (typically <1 cm in diameter) and reproductive organs (flowers and fruits) [37]. Litterfall samples were dried in a glasshouse (7 days, 60°C) before measuring their biomass with a precision scale. One litterfall measurement was discarded because of a freshly fallen climber fruit that biased the analysis (>7 g day^−1^). A carbon content of 42% for litterfall, standing leaf litter and woody debris was used [38].

### Soil respiration rates

Soil respiration rates were estimated following the methods reported in [15]. Briefly, we took measurements using an Infrared Gas Analyzer CARBOCAP GMP343 (Vaisala, Finland) and a self made chamber [39] at forty sites along the transect lines in May and June 2007. We measured nine times (seven day time (08:00 am to noon) and two night time (08:00 pm to 04:00 am) measurements) over five minute intervals.

### Statistical analysis

Our focus for the C balance is on the estimation of means and variabilities, so we present point estimates with standard deviations (SD; given as mean ± SD), standard errors (SEM; given as mean ± SEM), or confidence intervals (CI; given as mean with lower–upper bound) as appropriate. Standard deviations for the total sum of means are reported as the sum of the weighted variances, where the weighting is based on the relative contribution of the single mean and the number of replications (see Table S3).

Fixed effects estimates derived from meta-analytical methods were used for the across study comparison of forest types (selectively logged and unlogged) [40]. Mean differences (with 95% CI) were calculated with the metacont function from the *meta* package (version 1.6-1) for R 2.12.1 [41], using means, SD and replication (see Table S6). Inverse variance weighting was used for pooling the data.

Simple t-tests were performed for comparing components of the C balance between published literature estimates (mean ± SEM) and own measures (mean ± SEM). Differences were reported with the corresponding standard error of the difference (SED) (Table 2). Non-linear patterns in litterfall rates were examined by fitting a cubic polynomial regression to the log transformed response variable (see Fig. S1).

**Table 2:** Comparison of basal area, volume and selected stocks and fluxes related to C turnover processes.

	Unit	Logged	Unlogged	Difference	t	p
Basal area	m^2^ ha^−1^	25±0.8	29.9±0.7	4.9 (±1.1)	4.6	>0.01
– Dipterocarp	m^2^ ha^−1^	6.9±0.2	18.2±0.7	11.3 (±0.7)	15.5	>0.0001
– Pioneer	m^2^ ha^−1^	8.7±0.2	0.1±0.1	8.6 (±0.2)	38.5	>0.0001
Basal area SDW	m^2^ ha^−1^	6.0±0.3	5.6±0.5^a^	0.4 (±0.6)	0.7	ns
C components						
TAGC	Mg C ha^−1^	91.9±2.9	128±13.4	36.1 (±13.7)	2.6	>0.05
Downed woody debris	Mg C ha^−1^	20.6±8.8	70.6±9.5^a^	50.0 (±13.4)	3.7	>0.001
SDW	Mg C ha^−1^	8.7±3.5	6.5±1.1^a^	2.2 (±3.7)	0.6	ns
Fine root biomass	Mg C ha^−1^	0.95±0.1	0.85±0.02^b^	0.1 (±0.3)	0.3	ns
Flux sampling						
Fine litterfall rate	Mg C ha^−1^ yr^−1^	4.9±0.1	4.8±0.1^c^	0.1 (±0.1)	0.7	ns
Soil respiration rate	Mg C ha^−1^ yr^−1^	28.6±1.2	21.6±1.4^d^	7.0 (±1.9)	3.7	>0.001

Means ± SEM from selectively logged forest (Sabah Biodiversity Experiment) and unlogged forest sites (Danum Valley, except for soil respiration reported from Lambir Hills, Sarawak, North Borneo). Difference (± SED) is reported with the corresponding t-value and level of significance. SDW: Standing deadwood, TAGC: Total aboveground carbon.

a Gale 2000.

b Green *et al.* 2005.

c Burghouts *et al.* 1992.

d Katayama *et al.* 2009.

## Results

Based on our sampling of the Sabah Biodiversity Experiment plots, we estimated the total C stock in selectively logged areas of the Malua Forest Reserve as 167.9 Mg C ha^−1^±3.8 (SD). Total C can be split roughly 2/3 to 1/3 with aboveground stocks contributing 65% and belowground stocks 35%. We first present four main C stocks that contribute 97% to the C balance and then three minor C stocks (3%). Then we report the two C fluxes litterfall and heterotrophic soil respiration.

### C stocks in the Sabah Biodiversity Experiment area of the Malua Forest Reserve

**Figure 2:**
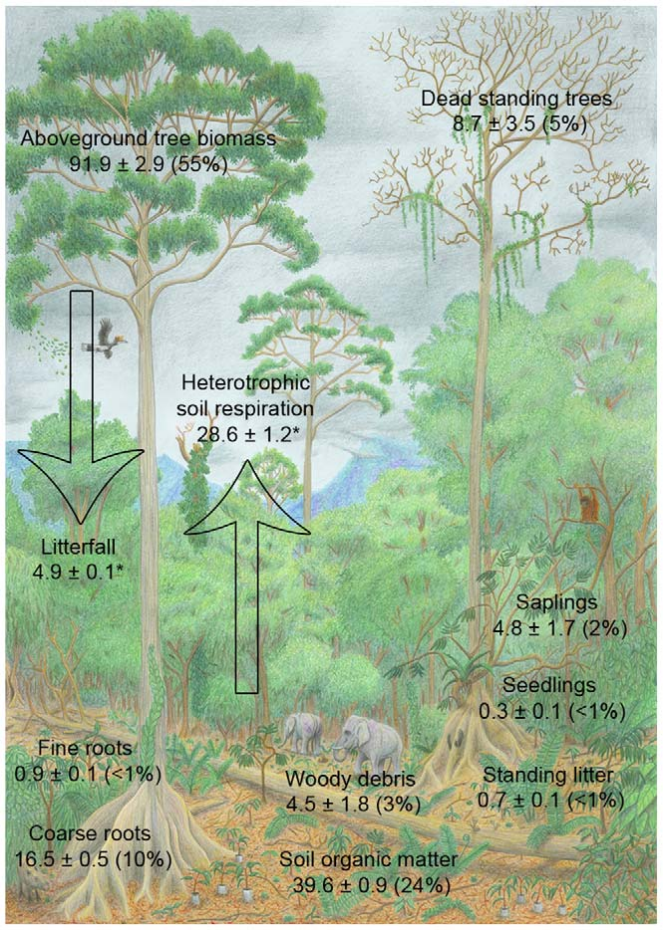
Pre-enrichment planting C balance for the Sabah Biodiversity Experiment. The area is located in selectively logged forest of the Malua Forest Reserve. All components are given as mean ± SEM Mg C ha^−1^, except for C fluxes (*), which are reported as Mg C ha^−1^ yr^−1^. Values in parenthesis indicate the percentage of single C stocks to total organic C (167.9 Mg C ha^−1^).

Major C stocks included total aboveground (TAGC: 55%; 91.9 Mg C ha^−1^±2.9 SEM) and belowground biomass in trees (TBGC: 10%; 16.5 Mg C ha^−1^±0.5 SEM), deadwood (DW: 8%; 13.2 Mg C ha^−1^±3.5 SEM) and soil organic matter (SOM: 24%; 39.6 Mg C ha^−1^±0.9 SEM). Minor stocks included understory vegetation (UV: 3%; 5.1 Mg C ha^−1^±1.7 SEM), standing litter (SL: <1%; 0.7 Mg C ha^−1^±0.1 SEM) and fine root biomass (FRB: <1%; 0.9 Mg C ha^−1^±0.1 SEM) (Fig. 2).

### Comparison of logged and unlogged forest: basal area, total aboveground tree biomass and C stocks

**Figure 3:**
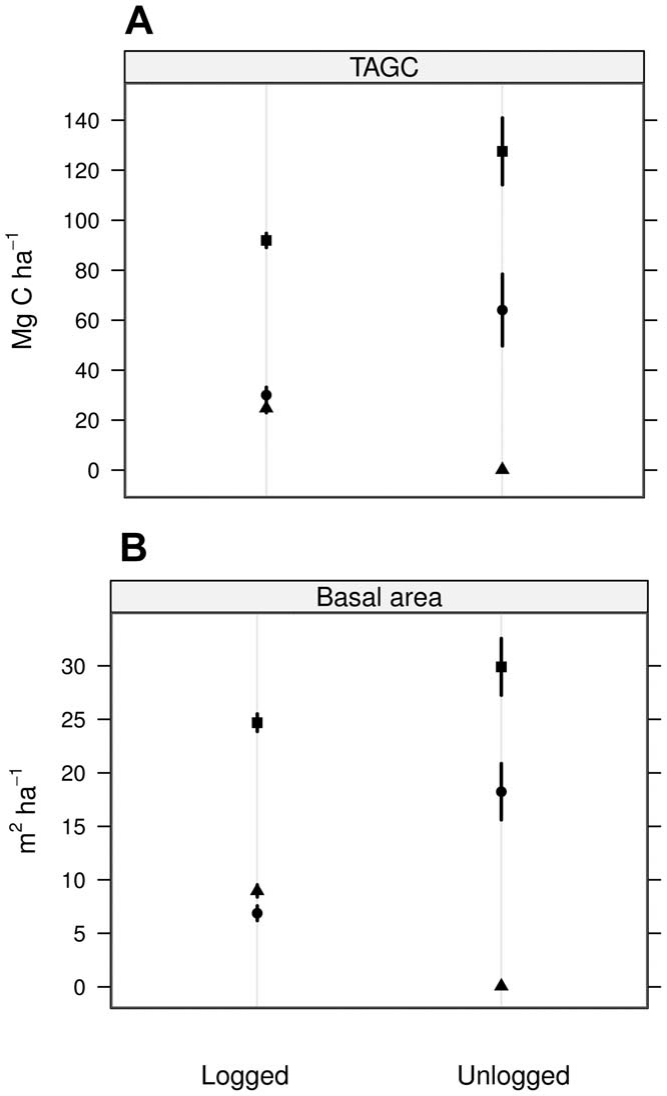
Total aboveground C stocks (TAGC) and basal area in selectively logged an unlogged forest. All species (square), dipterocarps (circle) and pioneer species only (triangle) (mean ± SEM; n = 4). Decreases in dipterocarp basal area but not in carbon stocks were compensated by the pioneers.

Total basal area of trees >10 cm DBH was significantly higher in unlogged primary forest at Danum Valley compared to the selectively logged forest of the Sabah Biodiversity Experiment in Malua (Fig. 3B and Table 2, Table S4 and S5). The observed difference was due to tree size rather than tree density, which was similar between both types (417 trees ha^−1^ for selectively logged versus 410 trees ha^−1^ for unlogged forest). To examine the impacts of logging, we separated total basal area into dipterocarp (the dominant component of unlogged primary forests and main target of logging) versus pioneer species, in our case principally *Macaranga pearsonii* and *Macaranga gigantea*, *Neolamarkia cadamba*, *Octomeles sumatrana* and *Duabanga moluccana*. Dipterocarps dominated the basal area in the unlogged forest (61%) but comprised only 28% of total basal area in the selectively logged forest. In contrast, pioneer species accounted for 35% of total basal area in selectively logged forest but less than 0.2% in the unlogged primary forest (Fig. 3B and Table 2).

**Figure 4:**
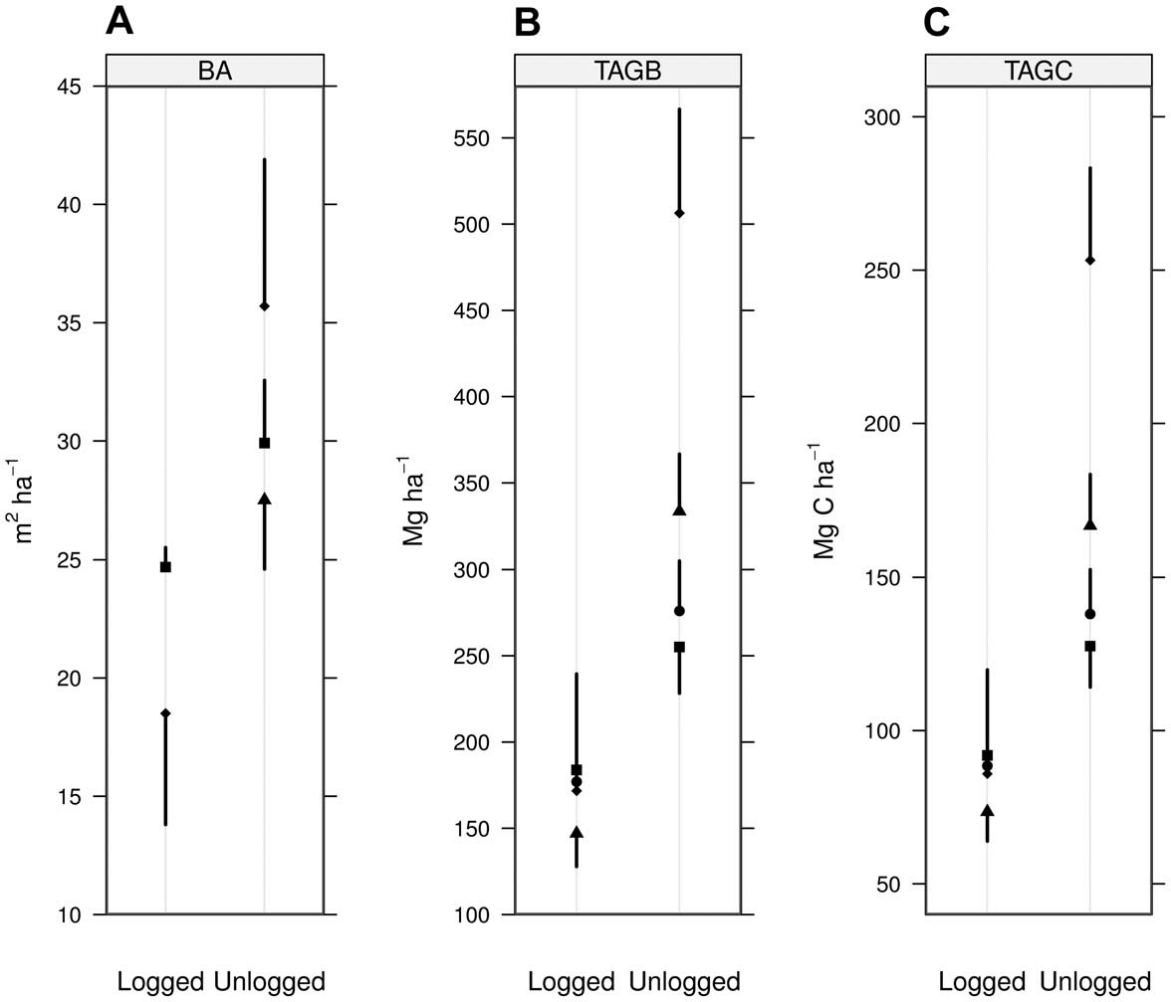
Across study comparison of basal area, aboveground biomass and C. Mean (plus or minus one SEM) are shown for basal area (m^2^ ha^−1^), total aboveground biomass (TAGB Mg ha^−1^), and total aboveground carbon (TAGC Mg C ha^−1^) assessed at Danum Valley Conservation Area and nearby selectively logged areas including the Sabah Biodiversity Experiment. Berry 2010 (circle): selectively logged measurements were taken 18 years after harvest; Pinard 1996 (triangle) and Tangki 2008 (diamond): 20 years after harvest; this study (quadrat): 22 years after harvest. All unlogged measurements are from Danum Valley Convervation Area except Pinard 1996 which were taken on unlogged Ulu Segama forest. Note that Berry 2010 (logged and unlogged) and Pinard 1996 (logged) did not specify basal area.

A combined weighted average for the four independent studies (Fig. 4B and Table S6) showed that tree aboveground biomass stocks (TAGB) were significantly lower (z = 6.4, trials = 4, p<0.0001) in selectively logged compared to unlogged primary forest (mean difference −115.7 Mg ha^−1^; with 95% CI −151.0 to −80.4). For the present study the mean difference (−71.3 Mg ha^−1^; with 95% CI −158.6 to −16.1) was in large part due to the bigger canopy trees (>90 cm DBH) that were missing in selectively logged forest (DBH range selectively logged forest: 10.0–84.3 cm; primary unlogged forest 10.1–170.3 cm). With a pre-logging dipterocarp timber volume of 180–216 m^3^ ha^−1^ and a mean wood density of 0.6 g cm^−3^ the total extractable wood was 108–130 Mg ha^−1^. Based on the observed mean difference (71.3 Mg ha^−1^) this indicates that 55–66% of the dipterocarp stock was exploited during selective logging.

Assuming that half of the biomass is stored C (see [8] for discussion), the across-study comparison predicts a C loss of −57.9 Mg C ha^−1^ (with 95% CI −75.5 to −40.2) for selectively logged forest in the region and a loss of −35.7 Mg C ha^−1^ (with 95% CI −79.3 to −8.1) for the Sabah Biodiversity Experiment in particular. This corresponds to 28% (with 95% CI 62% to 6%) net aboveground C loss 22 years after logging.

### Belowground biomass

Belowground coarse roots were assumed to contribute 10% to the total C balance based on literature values [27] and were not confirmed by measurement on site.

### Deadwood

In total a mean density of 48.6±10.2 (SEM) dead stems ha^−1^ was counted. Basal area and C stocks in dead standing trees were not found to significantly differ compared to unlogged primary forest at Danum Valley (Table 2).

### Soil organic matter

**Figure 5:**
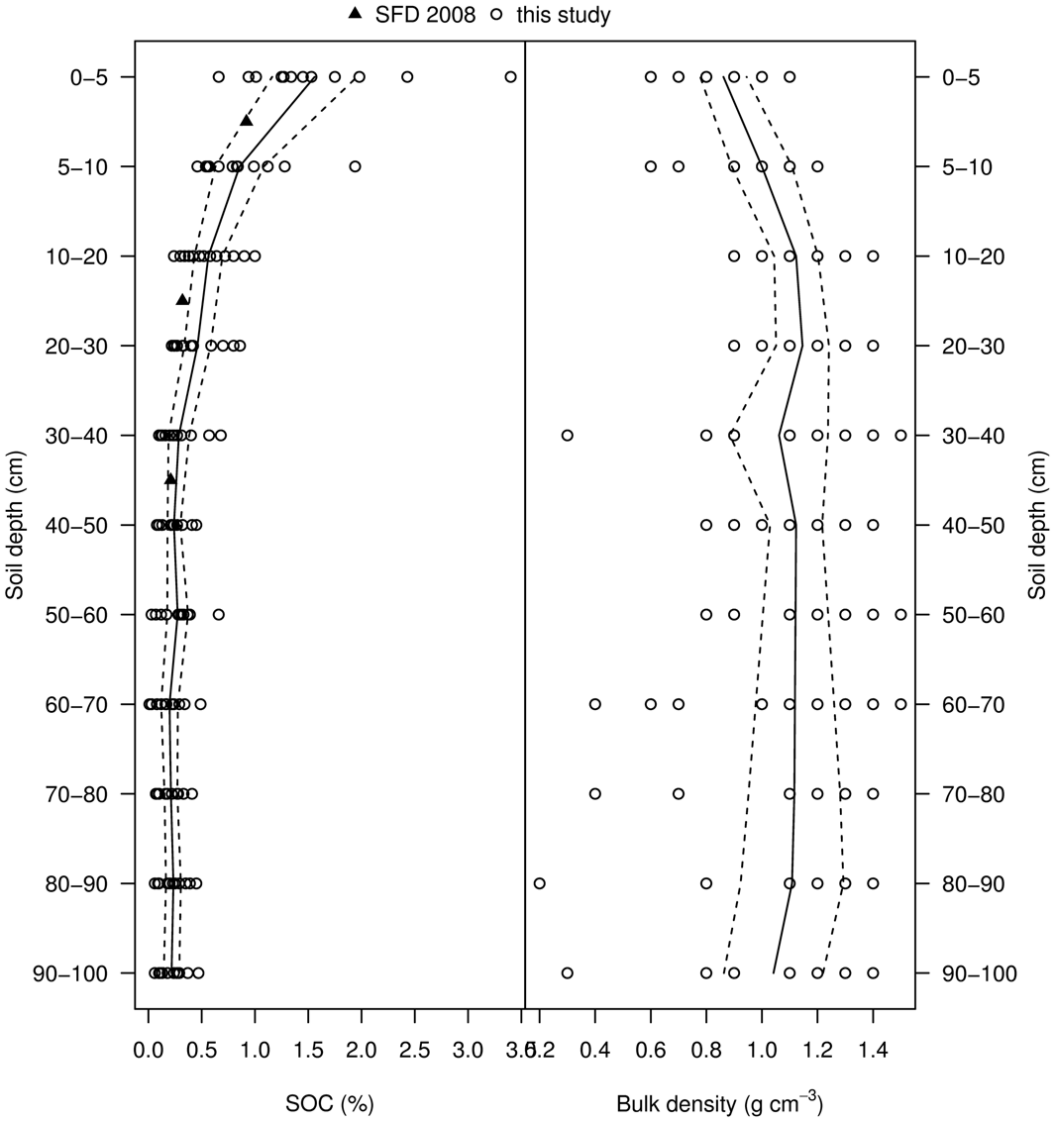
Soil C depth profile. Measured soil organic C (SOC) and bulk density with depth for the Sabah Biodiversity Experiment (Saner 2009). Overlayed points are soil organic C typical of the Kretam soil association (SFD 2008), which is found at Malua Forest Reserve. The line is a smoothed loess curve of mean SOC (±95% CI).

Soil C content in organic matter was relatively low across all sites (39.6 Mg C ha^−1^±0.9 SEM). Highest concentrations were found in the top 0.3 m (22.0 Mg C ha^−1^), in the sub-soil (0.3–1 m depth) figures decreased to 17.6 Mg C ha^−1^. Mean soil bulk density (1.1 g cm^−3^±0.1 SEM) was lower in the top soil (<0.1 m) of selectively logged forest (0.9 g cm^−3^±0.1 SEM) compared to 0.1 to 1 m depth (1.1 g cm^−3^±0.1 SEM) (Fig. 5 and Table S7).

### Understory vegetation

The contribution of sapling and seedling biomass to the total C balance was minor. Nevertheless, the density and diversity of the natural occuring dipterocarp saplings and seedlings are of interest for the forest rehabilitation efforts [18]. Total basal area of all dipterocarp saplings and seedlings was 0.46 m^2^ ha^−1^. A mean dipterocarp sapling and seedling density of 800±240 (SEM) ha^−1^ was measured for selectively logged forest. Dipterocarp saplings and seedlings of ten species were identified in a total area of 0.06 ha.

### Standing litter

Standing litter was also a minor contributor to the total C balance (Fig. 2). In particular downed woody debris was reported to be much higher in unlogged forest compared to our estimates from selectively logged forest (Table 2). A more extensive survey could lead to higher estimates for the contribution of woody debris to total C stocks.

### Fine root biomass

Despite the fact, that we took fine roots down to 5 cm soil depth only, our estimations were not found to significantly differ from more extensive surveys to 15 cm soil depth [42] (Table 2).

### Litterfall

**Figure 6:**
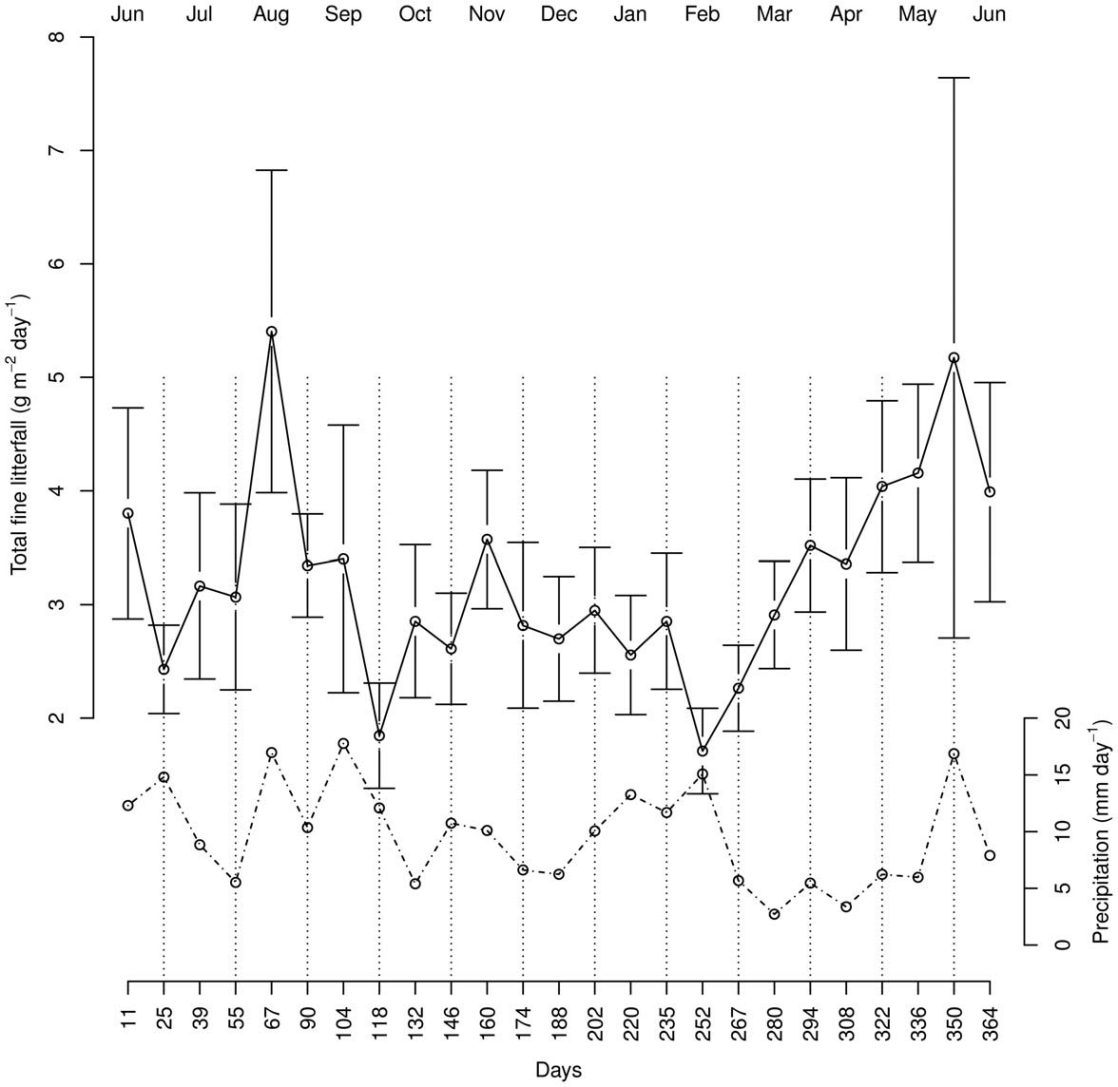
Monthly estimates of litterfall. Time series of total fine litterfall (solid line; mean±95% CI) and precipitation (dashed line; mean). Days indicate litter collections (n = 25) in 2006/07. Mean litterfall and rainfall were calculated over the fourteen days prior to collection.

Mean fine litterfall rate, including leaves, small twigs (<1 cm DBH) and reproductive organs was 4.9 Mg C ha^−1^ yr^−1^±0.1 (SEM). Considering leaf litter only, the total amount was 3.2 Mg C ha^−1^ yr^−1^±0.2 (SEM). Litterfall varied significantly between collection dates (n = 25) (Fig. 4). Peaks in litterfall occurred during the months of August 2006 (0.5 Mg C ha^−1^ month^−1^±0.1 SEM) and May 2007 (0.6 Mg ha^−1^ month^−1^±0.1 SEM). Lowest litterfall rates occurred in September 2006 (0.3 Mg ha^−1^ month^−1^±0.1 SEM) and February 2007 (0.2 Mg ha^−1^ month^−1^±0.1 SEM). There was no apparent seasonal trend in litterfall rates (Fig. 6) related to the wetter months from November to March and during June and July [14]. Litterfall rates from this study were not significantly different from reported estimates of 15 year old selectively logged forest [38] and unlogged forest (Table 2).

### Soil respiration rates

Overall soil respiration rate, measured over two months (May and June 2007) and extrapolated for one year were significantly higher than reported values from Pasoh Forest Reserve in West Peninsular Malaysia [43] or more recent estimates from Lambir, Sarawak [44] (Table 2). Future more extensive studies are likely to report lower estimates for yearly rates of soil respiration rates for this region.

## Discussion

The main finding of this study is that the biomass stock of the Sabah Biodiversity Experiment is 28% less than that of pristine forest even 22 years after logging. We estimate that 55–66% of the pre-logging dipterocarp stock was exploited by selective logging, resulting in significant C losses. Approximately 20% of the basal area in selectively logged forest was later occupied by fast growing pioneer trees, which contributed substantially less to the C stock. We also found that the density but not the diversity of the dipterocarp sapling and seedling stock was high [18]. In combination, these observations indicate that lowland dipterocarp rainforest that is disturbed by selective logging will lose desirable and specialized dipterocarp species, but that overall levels of diversity may be maintained by competitive release of early successional species following disturbance.

Recovery of exploitation from conventional logging may take half a century as reported by findings from French Guiana [45]. However, restoration and management practices that increase dipterocarp recruitment and basal area have the potential to increase C stocks of selectively logged forest and also may accelerate the return to pre-logging levels.

### C stock calculation and potential biases

A total C stock of 167.9 Mg C ha^−1^±3.8 (SD) was measured for selectively logged forest in this study. This is substantially lower than reported elsewhere [18], [46], most likely because of the different methodology used to calculate aboveground biomass. Estimates of the Ulu Segama Forest Reserve, adjacent to the area under study report 261 Mg C ha^−1^, including total biomass of 200 Mg C ha^−1^±10 SEM, woody debris and litter (28 Mg C ha^−1^) and soil organic matter (33 Mg C ha^−1^) for selectively logged forest [46]. Several assumptions are likely to induce large biases when estimating C stocks, including: site comparability, plot size, DBH measurement, selection of the DBH-biomass regression model, the belowground estimation of biomass and the overall C content in trees, and the C content in soil organic matter. In particular the three major components aboveground tree biomass, coarse root biomass and soil organic matter covered 89% of organic C stocks and should be estimated with greater precision.

### Site comparability

Here we assumed that the Malua Forest Reserve and the primary forest of Danum Valley Conservation Area were comparable before logging to infer the impacts of selective logging. Similarity of the two areas (prior to selective logging) is supported by several lines of evidence. First, the edaphic conditions are comparable including elevation, topography, parent material and soil type (Table 1 and Table S1). Yayasan Sabah (the Sabah Foundation) estimated similar volumes of extractable timber for the two sites at Danum and Malua (Table 1). Therefore, while we cannot be sure that the differences reported here are entirely due to selective logging previous reports suggest the areas were very initially similar and suggest that the differences can be largely attributed to timber extraction. The difference is, not surprisingly most likely due to the missing large trees (>90 cm DBH), and in particular the missing large dipterocarps in selectively logged forest.

### Plot size

Standing TAGB and standing deadwood was sampled based on a total sampling effort of 1 ha in both forest types. Based on analysis from La Selva (Costa Rica) [47] and Barro Colorado Island (Panama) [48] we assume that the associated sampling error is high. We therefore included estimates from the region to present an across-study loss in C stocks of −57.8 Mg C ha^−1^ (with 95% CI −75.5 to −40.2) for selectively logged forest in the region.

### DBH measurement and DBH-AGB regression model

Major differences in reported TAGC stocks across studies can be also accounted to the DBH measurement and the selection of the DBH-AGB regression model. Here we measured DBH above buttresses which may substantially underestimate basal area of large trees by ignoring the area occupied at the base [47]. However, basal area estimations are comparable to those published previously in selectively logged and unlogged forest [49], [50].

The quality of the allometric estimates may have led to the largest error propagation, in particular since it was applied to both, selectively logged and unlogged forest. In contrast to other studies [18], [46], [49], [51]–[52] we used a more recent DBH-AGB regression model as a standard methodology [22], which resulted in lower biomass C stocks (Fig. 4). For the study site the use of the more recent methodology resulted in 12% less difference between selectively logged and unlogged forest sites compared to an earlier estimation based on older methodologies (28% instead of 40%) [18]. Previous allometric equations [23]–[26] estimated the aboveground tree biomass at 357–517 Mg ha^−1^. Hence, we assume that the presented estimate is conservative.

### Belowground estimation of biomass

The estimation of root biomass, which is supposedly the second largest live store of C in a tropical forest, was calculated with a BGB/AGB ratio of 0.18 [27]. This is only slightly higher compared to the one used in previous studies of the region (0.17) [46].

### Overall C content in trees and soil organic matter

Another potential source for bias are general assumptions about the C content. The assumption that half of the aboveground and belowground biomass is C was challenged by recent results from Barro Colorado Island (Panama). They report C content from 41.9–51.6% among species and a mean C content of 47.4±2.5% SD. Based on their measured mean C content the biomass C stocks in the present study would decrease by 7.5 Mg C ha^−1^ (6%) [53].

Similar limitations apply to the derivation of C content in soil organic matter. It was measured down to 1 m soil depth and is based on the Walkley-Black method, a wet chemical analysis that may underestimate total content [54]. Future studies may therefore report higher estimates for the region.

### C fluxes and potential biases

Measured stocks and fluxes in selectively logged forest that are directly related to C turnover rates but also to forest disturbance include standing deadwood and coarse woody debris, fine root biomass, litterfall and soil respiration rates. Of these, standing deadwood, fine root biomass and litterfall were found to be not significantly different from previous reports of unlogged forest at Danum Valley. This indicates that apart from the lower C stocks in 22 years old selectively logged forest, at least some ecosystem processes could be maintained at the rate of primary forests with regard to nutrient and C turnover (Table 2). The example of fine litterfall rates, as a proxy for net primary productivity, indicates that such processes are likely to be non-linear (Fig. S1) and may also be affected by potential time lag in the response of decomposition processes. The observed peak in fine litterfall rate in drier periods could suggest that desiccation results in increased leaf abscission to reduce evapotranspiration. On the other hand, heavy rainfall appears to also result in increased litterfall. Due to the temporal limitations of the study (1 year) it is difficult to draw any firm conclusion about the correlation of seasonality in litterfall rates and rainfall, such as presented in a recent survey with >10 year data [36]. This may especially apply to dipterocarp-dominated forests, which are characterized by supra-annual patterns such as for example mast fruiting events. Temporal limitations also apply to the estimated soil respiration rate which is a measure of heterotrophic ecosystem respiration. It is derived from a limited dataset and suggests that a more extensive spatial and temporal sampling may result in lower figures, such as the estimate reported from Lambir Hills, Sarawak (North Borneo) (Table 2) [44].

In summary, more extensive research on the main C stocks and the fluxes, including wood NPP and autotrophic respiration should be undertaken to draw a more complete picture of the C balance of selectively logged forest.

### Implications for forest management and restoration

Based on the reported findings we argue that silvicultural treatments may have the potential to accelerate the recovery of dipterocarp C stocks to pre-logging levels. Nabuurs and Mohren [55] estimated on the basis of model outputs that approximately 80 Mg C ha^−1^ can be stored additionally in the short term (over several decades) by enrichment line planting which is higher than our weighted average for the region (57.8 Mg C ha^−1^) and close to the site specific estimate of the Sabah Biodiversity Experiment (71.3 Mg C ha^−1^). Calculating with a price of 14 € per ton of CO_2_ (as currently traded on the European Energy Exchange market) reveals that the C sequestration potential by enrichment planting in selectively logged forest could achieve a market value as high as 2,065–3,879 € ha^−1^ for a project duration of approximately 60 years. Compared to the profitability of converting selectively logged forest into palm oil plantations (2,817–7,075 € over a 30 year period [56]) the revenue from C credits is still lower. A higher price for C credits could be achieved if forest rehabilitation in developing and emerging countries is accepted under the trading scheme of the CDM (Clean Development Mechanism) [57]. This is currently not the case for the guideline of the Kyoto protocol but is a goal of REDD+, which may raise international funds for tropical forest restoration [6], thus helping to close the potential funding shortfall [58]. It emphasizes that forest management in the tropics should focus on the multiple benefits that forests can provide. For example, linking CO_2_ offsetting with other ecosystem functions and with biodiversity could potentially allow for a comprehensive approach towards managing forests for mitigating climatic effects and conserve biodiversity and ecosystem services [10], [51], [52].

## Supporting Information

Figure S1 **Non-linear relationship between litterfall and rainfall.**
(PDF)

Table S1 **Soil characteristics of Danum Valley and Malua Forest Reserve.**
(DOC)

Table S2 **Tree species of the selectively logged forest.**
(DOC)

Table S3 **Carbon balance of the selectively logged forest.**
(DOC)

Table S4 **Overview of most important tree families and species in unlogged forest.**
(DOC)

Table S5 **Overview of most important tree families and species in selectively logged forest.**
(DOC)

Table S6 **Study survey from in and around Danum Valley Conservation Area.**
(DOC)

Table S7 **Carbon content survey in soil organic matter.**
(DOC)
