# Supplementary material for: Carbon Stocks and Fluxes in Tropical Lowland Dipterocarp Rainforests in Sabah, Malaysian Borneo
Source: PLoS One. 2012 Jan 3;7(1):e29642. doi: 10.1371/journal.pone.0029642 (PMC3250468; doi:10.1371/journal.pone.0029642)

Figure S1 A generalized additive model (GAM) with 3df regression spline (y-axis) term was fit to show the 3rd degree (cubic) relationship between litterfall and mean rainfall.

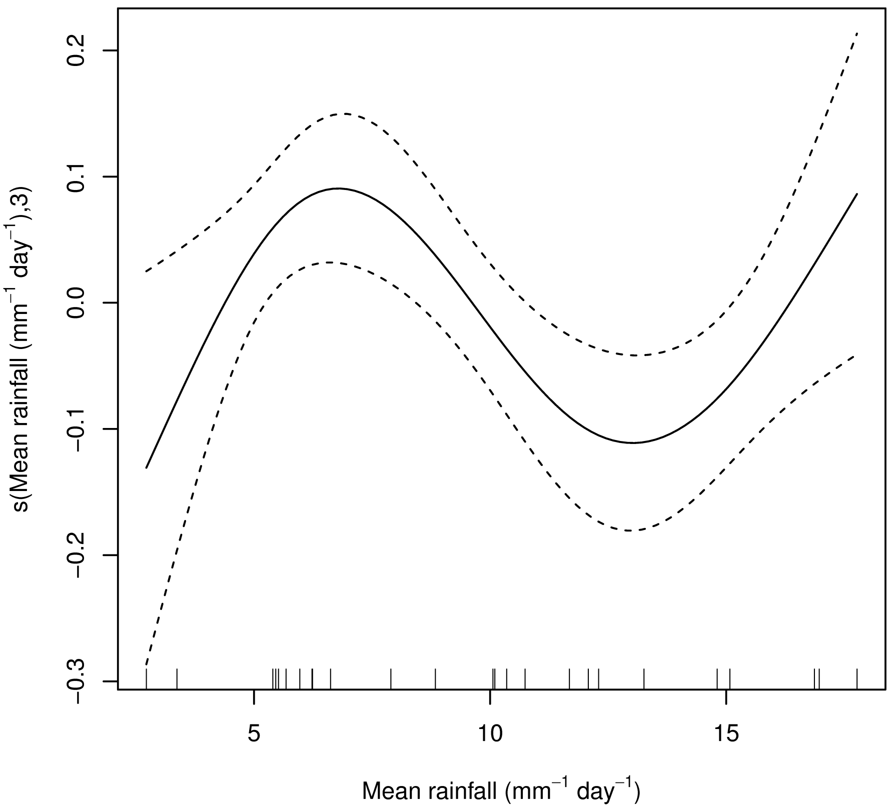

Supplement: Figure S1 — Non-linear relationship between litterfall and rainfall. (PDF) [file pone.0029642.s001.pdf]
