# Supplementary material for: Carbon Stocks and Fluxes in Tropical Lowland Dipterocarp Rainforests in Sabah, Malaysian Borneo
Source: PLoS One. 2012 Jan 3;7(1):e29642. doi: 10.1371/journal.pone.0029642 (PMC3250468; doi:10.1371/journal.pone.0029642)
Supplement: Table S1 — Soil characteristics of Danum Valley and Malua Forest Reserve. (DOC) [file pone.0029642.s002.doc]

Table S1 Characteristics of the Bang soil association sampled in Danum Valley and the Kretam soil association sampled in the Malua Forest Reserve at three different soil depths (cm).

|  | **Bang** | | |  | Kretam | | |
| --- | --- | --- | --- | --- | --- | --- | --- |
| **Depth** | **0-15** | **15-30** | **30-45** | **Depth** | **0-15** | **15-30** | **30-45** |
| **pH** | 4.1 | 4.31 | 4.36 | **pH** | 5.4 | 4.66 | 4.84 |
| **Organic C** | 1.5 | 0.79 | 0.46 | **Organic C** | 0.92 | 0.32 | 0.21 |
| **Mg** | 0.94 | 0.73 | 0.8 | **Mg** | 2.2 | 2.71 | 4.33 |
| **Ca** | 1.87 | 0.93 | 0.3 | **Ca** | 6.26 | 5.1 | 5.93 |
| **Na** | 0.03 | 0.03 | 0.03 | **Na** | 0.03 | 0.04 | 0.03 |
| **K** | 0.19 | 0.2 | 0.06 | **K** | 0.17 | 0.23 | 0.25 |
| **CEC** | 15.93 | 16.47 | 13.81 | **CEC** | 9.37 | 10.51 | 15.59 |
| **Clay** | 55 | 44 | 49 | **Clay** | 29 | 35 | 32 |
| **Silt** | 15 | 21 | 24 | **Silt** | 20 | 22 | 31 |
| **Sand** | 30 | 35 | 28 | **Sand** | 51 | 43 | 30 |
