# Supplementary material for: Carbon Stocks and Fluxes in Tropical Lowland Dipterocarp Rainforests in Sabah, Malaysian Borneo
Source: PLoS One. 2012 Jan 3;7(1):e29642. doi: 10.1371/journal.pone.0029642 (PMC3250468; doi:10.1371/journal.pone.0029642)
Supplement: Table S2 — Tree species of the selectively logged forest. (DOC) [file pone.0029642.s003.doc]

Table **S2** Trees (>10 cm DBH) of the selectively logged forest of the Sabah Biodiversity Experiment identified to species or genus level. **Wood Density**: From the World Agroforestry Centre Wood Density Database (<http://www.worldagroforestry.org/sea/Products/AFDbases/WD/Index.htm>). **IUCN Status** (2011): Critical (CR), Vulnerable (VU), Endangered (EN).

| **Family** | **Botanical name** | **Wood Density (g cm^-3^)** | **IUCN Status** |
| --- | --- | --- | --- |
| Lauraceae | *Actinodaphne sp.* | 0.62 |  |
| Theaceae | *Adinandra myreniura* | 0.7 |  |
| Meliaceae | *Aglaia macrocarpa* Miq. | 0.69 |  |
| Meliaceae | *Aglaia odoratissima* Bl. | 0.89 |  |
| Meliaceae | *Aglaia squamulosa* King | 0.79 |  |
| Alangiaceae | *Alangium griffithii* Harms | 0.87 |  |
| Alangiaceae | *Alangium javanicum* (Bl) Wangerin | 0.88 |  |
| Lauraceae | *Alseodaphne sp.* | 0.63 |  |
| Euphorbiaceae | *Aporusa accuminatissima* Merr. | 0.73 |  |
| Euphorbiaceae | *Aporusa elmerii* Merr. | 0.73 |  |
| Euphorbiaceae | *Aporusa grandistipula* Merr. | 0.73 |  |
| Thymelaeaceae | *Aquilaria malaccensis* Lamk. | 0.4 | VU |
| Leguminosae | *Archidendron sp.* | 0.61 |  |
| Myrsinaceae | *Ardisia sp.* | 0.51 |  |
| Moraceae | *Artocarpus anisophyllus* Miq. | 0.74 |  |
| Chrysobalanaceae | *Atuna cordata* Cockburn ex Prance | 0.84 | VU |
| Meliaceae | *Azadiracta excelsa* Jacobs | 0.6 |  |
| Euphorbiaceae | *Baccaurea stipulata* J.J. Smith | 0.79 |  |
| Euphorbiaceae | *Baccaurea tetandra* | 0.8 |  |
| Lecythidaceae | *Barringtonia macrostachya* Jack | 0.65 |  |
| Lauraceae | *Beilschmiedia sp.* | 0.62 |  |
| Euphorbiaceae | *Blumeodendron tokbrai* J.J. Smith | 0.65 |  |
| Tiliaceae | *Brownlowia peltata* Benth. | 0.6 |  |
| Anacardiaceae | *Buchanania sessilifolia* Bl. | 0.51 |  |
| Labiatae | *Callicarpa sp.* | 0.41 |  |
| Guttiferae | *Calophyllum sp.* | 0.68 |  |
| Theaceae | *Camellia sp.* | 0.62 |  |
| Burseraceae | *Canarium denticulatum* Bl. | 0.63 |  |
| Burseraceae | *Canarium odontophyllum* Miq. | 0.64 |  |
| Fagaceae | *Castanopsis sp.* | 0.76 |  |
| **Table S2 continued...** |  |  |  |
| Olacaceae | *Chionanthus sp.* | 0.67 |  |
| Meliaceae | *Chisocheton sp.* | 0.71 |  |
| Lauraceae | *Cinnamomum sp.* | 0.43 |  |
| Euphorbiaceae | *Cleistanthus myrianthus* Kurz | 0.72 |  |
| Euphorbiaceae | *Cleistanthus paxii* Jabl. | 0.69 |  |
| Euphorbiaceae | *Clorophyllum wallichinum* | 0.83 |  |
| Guttiferae | *Cratoxylum sp.* | 0.53 |  |
| Leguminosae | *Crudia reticulata* Merr. | 0.93 |  |
| Lauraceae | *Cryptocaria sp.* | 0.61 |  |
| Leguminosae | *Cynometra inaequifolia* Knaap-v. M. | 0.96 | VU |
| Burseraceae | *Dacryodes rostrata* Bl. | 0.66 |  |
| Lauraceae | *Dehassia sp.* | 0.74 |  |
| Urticaceae | *Dendrocnide elliptica* Chew | 0.62 |  |
| Leguminosae | *Dialium indum* L. | 0.98 |  |
| Dilleniaceae | *Dillenia excelsa* Jack | 0.87 |  |
| Sapindaceae | *Dimocarpus dentatus* | 0.87 |  |
| Sapindaceae | *Dimocarpus longan* Lour. | 0.87 |  |
| Sapindaceae | *Dimocarpus longan* Lour. | 0.87 |  |
| Ebenaceae | *Diospyros elliptifolia* Merr. | 0.94 |  |
| Ebenaceae | *Diospyros macrocarpa* L. | 1.03 |  |
| Ebenaceae | *Diospyros muricata* L. | 1.03 |  |
| Rubiaceae | *Diplospora sp.* | 0.62 |  |
| Dipterocarpaceae | *Dipterocarpus caudiferus* Merr. | 0.72 |  |
| Dracaenaceae | *Dracaena angustifolia* | 0.62 |  |
| Anacardiaceae | *Dracontomelon sp.* | 0.61 |  |
| Dipterocarpaceae | *Dryobalanops lanceolata* Burck | 0.81 | EN |
| Euphorbiaceae | *Drypetes sp.* | 0.88 |  |
| Sonneratiaceae | *Duabanga moluccana* Bl. | 0.39 |  |
| Bombacaceae | *Durio grandiflorus* Mast. | 0.63 |  |
| Meliaceae | *Dysoxylum sp.* | 0.71 |  |
| Elaeocarpaceae | *Elaeocarpus stipularis* Bl. | 0.57 |  |
| Lauraceae | *Endiandra rubescens* Miq. | 0.79 |  |
| Euphorbiaceae | *Endorspermum diadenum* Airy Shaw | 0.48 |  |
| Euphorbiaceae | *Endorspermum peltatum* Merr. | 0.38 |  |
| **Table S2 continued...** |  |  |  |
| Annonaceae | *Enicosanthum sp.* | 0.66 |  |
| Myrtaceae | *Eugenia sp.* | 0.78 |  |
| Lauraceae | *Eusideroxylon zwageri* Teijs. & Binn. | 0.99 | VU |
| Loganiaceae | *Fagraea volubilis* Wall. | 0.79 |  |
| Moraceae | *Ficus truebii* | 0.47 |  |
| Moraceae | *Ficus verigata* | 0.28 |  |
| Guttiferae | *Garcinia sp.* | 0.91 |  |
| Euphorbiaceae | *Glochidion rubrum* Bl. | 0.8 |  |
| Anacardiaceae | *Gluta wallichii* D. Hou | 0.73 |  |
| Sapindaceae | *Guioa pubescens* | 0.68 |  |
| Myristicaceae | *Gymnacranthera sp.* | 0.72 |  |
| Proteaceae | *Helicia sp.* | 0.65 |  |
| Sterculiaceae | *Heritiera elata* Ridley | 0.92 |  |
| Dipterocarpaceae | *Hopea beccariana* Burck | 0.75 | CR |
| Dipterocarpaceae | *Hopea nervosa* King | 0.75 | CR |
| Flacourtiaceae | *Hydnocarpus borneensis* Sleumer | 0.82 |  |
| Flacourtiaceae | *Hydnocarpus kunstleri* | 0.82 |  |
| Flacourtiaceae | *Hydnocarpus polypetala* | 0.82 |  |
| Flacourtiaceae | *Hydnocarpus sumatrana* | 0.82 |  |
| Flacourtiaceae | *Hydnocarpus woodii* Merr. | 0.82 |  |
| Simaroubaceae | *Irvingia malayana* Oliv. ex Benn. | 1.07 |  |
| Ixonanthaceae | *Ixonanthes sp.* | 0.83 |  |
| Tiliaceae | *Jarandersonia rinoreoides* | 0.62 |  |
| Guttiferae | *Kayea oblongifolia* | 0.62 |  |
| Apocynaceae | *Kibatalea arborea* | 0.62 |  |
| Myristicaceae | *Knema sp.* | 0.69 |  |
| Euphorbiaceae | *Koilodepas longifolium* Hook. | 0.99 |  |
| Euphorbiaceae | *Koilodepas pectinatum* | 0.99 |  |
| Meliaceae | *Lansium sp.* | 0.84 |  |
| Leeaceae | *Leea indica* Merr. | 0.51 |  |
| Fagaceae | *Lithocarpus sp.* | 0.81 |  |
| Lauraceae | *Litsea caulocarpa* | 0.56 |  |
| Lauraceae | *Litsea firma* Hk. | 0.58 |  |
| Celastraceae | *Lophopetalum javanicum* Zoll. | 0.55 |  |
| **Table S2 continued...** |  |  |  |
| Rubiaceae | *Ludecia bornensis* | 0.67 |  |
| Euphorbiaceae | *Macaranga conifera* Muell. Arg. | 0.4 |  |
| Euphorbiaceae | *Macaranga gigantea* Muell. Arg. | 0.39 |  |
| Euphorbiaceae | *Macaranga hypoleuca* Muell. Arg. | 0.32 |  |
| Euphorbiaceae | *Macaranga pearsonii* Merr. | 0.36 |  |
| Sapotaceae | *Madhuca malaccensis* H.J. Lam | 0.76 |  |
| Magnoliaceae | *Magnolia candolii* H.Keng | 0.63 |  |
| Magnoliaceae | *Magnolia gigantea* | 0.61 |  |
| Magnoliaceae | *Magnolia gigantifolia* | 0.61 |  |
| Euphorbiaceae | *Mallotus muticus* Airy Shaw | 0.5 |  |
| Euphorbiaceae | *Mallotus penangensis* Muell. Arg. | 0.6 |  |
| Euphorbiaceae | *Mallotus phillippensis* Muell. Arg. | 0.71 |  |
| Euphorbiaceae | *Mallotus stipularis* Airy Shaw | 0.6 |  |
| Anacardiaceae | *Mangifera sp.* | 0.61 |  |
| Cornaceae | *Mastixia sp.* | 0.58 |  |
| Anacardiaceae | *Melanochylla sp.* | 0.68 |  |
| Rutaceae | *Melicope luna-akenda* T.G. Hartley | 0.42 |  |
| Sabiaceae | *Meliosma pinnata* Maxim. | 0.39 |  |
| Sabiaceae | *Meliosma pinnata* Maxim | 0.39 |  |
| Sabiaceae | *Meliosma sumatrana* Walp. | 0.5 |  |
| Melastomataceae | *Memecylon sp.* | 0.96 |  |
| Tiliaceae | *Microcos crassifolia* Burret | 0.57 |  |
| Sapindaceae | *Mischocarpus sp.* | 0.93 |  |
| Myristicaceae | *Myristica sp.* | 0.54 |  |
| Rubiaceae | *Nauclea subdita* Steud. | 0.59 |  |
| Bombacaceae | *Neesia sp.* | 0.56 |  |
| Rubiaceae | *Neolamarckia cadamba* Bosser | 0.43 |  |
| Rubiaceae | *Neonauclea artocarpoiedes* Merr. | 0.71 |  |
| Rubiaceae | *Neonauclea gigantea* Merr. | 0.75 |  |
| Rubiaceae | *Nephelium rambutan* | 0.85 |  |
| Olacaceae | *Ochanostachys amentacea* Mast | 0.91 |  |
| Datiscaceae | *Octomeles sumatrana* Miq. | 0.33 |  |
| Sapotaceae | *Palaquium sp.* | 0.65 |  |
| **Table S2 continued...** |  |  |  |
| Sapindaceae | *Paranephelium xestophyllum* Miq. | 1.01 |  |
| Dipterocarpaceae | *Parashorea malaanonan* Merr*.* | 0.53 | CR |
| Dipterocarpaceae | *Parashorea tomentella* Meijer | 0.4 |  |
| Sapotaceae | *Payena sp.* | 0.55 |  |
| Leguminosae | *Peltophorum racemosum* Merr. | 0.72 |  |
| Tiliaceae | *Pentace adenophora* Kost. | 0.64 |  |
| Tiliaceae | *Pentace laxiflora* Merr. | 0.64 |  |
| Rubiaceae | *Pleiocarpidia sandakanica* Brem. | 0.62 |  |
| Annonaceae | *Polyalthia obliqua* | 0.73 |  |
| Annonaceae | *Polyalthia sumatrana* Kurz | 0.73 |  |
| Sapindaceae | *Pometia pinnata* Forst. | 0.75 |  |
| Melastomataceae | *Pternandra coerulescens* Jack | 0.6 |  |
| Sterculiaceae | *Pterospermum elongatum* Korth | 0.52 |  |
| Euphorbiaceae | *Ptychopyxis kingii* Miq. | 0.65 |  |
| Flacourtiaceae | *Ryparosa sp.* | 0.69 |  |
| Annonaceae | *Sagerae lanceolata* Miq. | 0.73 |  |
| Meliaceae | *Sandoricum koetjape* Merr. | 0.42 |  |
| Burseraceae | *Santiria tomentosa* Bl. | 0.65 |  |
| Leguminosae | *Saraca declinata* Miq. | 0.6 |  |
| Sterculiaceae | *Scaphium sp.* | 0.66 |  |
| Dipterocarpaceae | *Shorea agamii* Ashton | 0.67 | EN |
| Dipterocarpaceae | *Shorea atrinervosa* Sym. | 0.95 |  |
| Dipterocarpaceae | *Shorea faguetiana* Heim | 0.55 | EN |
| Dipterocarpaceae | *Shorea falciferoides* Foxw. | 0.89 |  |
| Dipterocarpaceae | *Shorea fallax* Meijer | 0.63 |  |
| Dipterocarpaceae | *Shorea gibbosa* Brandis | 0.61 |  |
| Dipterocarpaceae | *Shorea johorensis* Foxw. | 0.51 | CR |
| Dipterocarpaceae | *Shorea leprosula* Miq. | 0.56 | EN |
| Dipterocarpaceae | *Shorea leptoderma* Meijer | 0.96 | CR |
| Dipterocarpaceae | *Shorea macroptera* Dyer | 0.48 | VU |
| Dipterocarpaceae | *Shorea parvifolia* Dyer | 0.56 | VU |
| Dipterocarpaceae | *Shorea parvistipulata* Heim | 0.31 |  |
| Dipterocarpaceae | *Shorea pauciflora* King | 0.66 | EN |
| Dipterocarpaceae | *Shorea superba* Sym. | 0.9 |  |
| Leguminosae | *Sindora sp.* | 0.68 |  |
| **Table S2 continued...** |  |  |  |
| Euphorbiaceae | *Spathiostemon javensis* | 0.62 |  |
| Icacinaceae | *Stemonurus scorpioides* Becc. | 0.62 |  |
| Leguminosae | *Sympetalandra borneensis* Stapf | 0.79 |  |
| Symplocaceae | *Symplocos fasciculata* Zoll. | 0.43 |  |
| Verbenaceae | *Teijsmanniodendron bogoriense* Koord. | 0.45 |  |
| Verbenaceae | *Teijsmanniodendron pteropodum* Bakh. | 0.47 |  |
| Combretaceae | *Terminalia citrina* Roxb. | 0.83 |  |
| Rubiaceae | *Urophyllum sp.* | 0.62 |  |
| Dipterocarpaceae | *Vatica albiramis* van Slooten | 0.67 |  |
| Dipterocarpaceae | *Vatica dulitensis* Sym. | 0.82 |  |
| Verbenaceae | *Vitex sp.* | 0.68 |  |
| Meliaceae | *Walsura pinnata* Hassk. | 1.05 |  |
| Polygalaceae | *Xanthophyllum flavecescens* | 0.81 |  |
| Rhamnaceae | *Zizyphus angustifolius* Miq. | 0.81 |  |
