# Supplementary material for: Carbon Stocks and Fluxes in Tropical Lowland Dipterocarp Rainforests in Sabah, Malaysian Borneo
Source: PLoS One. 2012 Jan 3;7(1):e29642. doi: 10.1371/journal.pone.0029642 (PMC3250468; doi:10.1371/journal.pone.0029642)
Supplement: Table S3 — Carbon balance of the selectively logged forest. (DOC) [file pone.0029642.s004.doc]

Table S3 Carbon balance of the Sabah Biodiversity Experiment. Mean (± SEM) is presented for main and minor C stocks and fluxes. Mean (± SD) is presented for the total sum of means (in bold), where the variance was calculated by a weighted estimate: , where k = # of components, w_i_ = mean of component / sum of means, s_i_^2^ = variance, n_i_= number of observations. C: Carbon. Note that coarse root biomass (TBGB) is reported based on a BGB/AGB ratio of 0.18. For further details see main text.

|  | **Range** | **Sample Size  (N)** | **Biomass  (Mg ha^-1^)** | **C Content  (%)** | **C  (Mg ha^-1^)** | **C (%)** | **Source** |
| --- | --- | --- | --- | --- | --- | --- | --- |
| **Main stocks** |  |  | **244.7 (± 10.9)** |  | **161.2 (± 5.4)** |  |  |
| TAGB | >10cm DBH | 4 transect lines;  1 ha | 183.8 (± 5.8) | 0.5 | 91.9 (± 2.9) | 55 | Basuki et al. 2009 |
| TBGB | >5mm diameter | 4 transect lines | 33.0 (± 1.0) | 0.5 | 16.5 (± 0.5) | 10 | Niiyama et al. 2010 |
| Dead standing trees |  | 4 transect lines;  1 ha | 17.3 (± 7.0) | 0.5 | 8.7 (± 3.5) | 5 | Own data, wood density estimate Delaney (1998) |
| Woody debris |  | 24 (each 25 m^2^ quadrate) | 10.6 (± 4.4) | 0.42 | 4.5 (± 1.8) | 3 | Own data; C content (Burghouts et al. 1992) |
| Soil organic matter | 1m depth | 396 (12 plots, 11 layers, 3 replicates) |  | 0.27 - 0.99 | 39.6 (± 0.9) | 24 | Own data |
| **Minor Stocks** |  |  | **13.6 (± 5.7)** |  | **6.7 (± 2.9)** |  |  |
| Saplings | >2m height, <10 cm DBH | 24 (each 25 m^2^ quadrate) | 9.5 (± 3.4) | 0.5 | 4.8 (± 1.7) | 2 | Own data |
| Seedlings | <2m height |  | 0.6 (± 0.2) | 0.5 | 0.3 (± 0.1) | <1 | Own data |
| Standing litter |  | 24 (each 25 m^2^ quadrate) | 1.6 (± 0.3) | 0.42 | 0.7 (± 0.1) | <1 | Own data; C content (Burghouts et al. 1992) |
| Fine root | (≤2 mm diameter)  0–5cm depth (topsoil) | 24 (each 25 m^2^ quadrate) | 1.9 (± 0.2) | 0.5 | 0.9 (± 0.1) | <1 | Own data |
|  |  |  |  |  |  |  |  |
| **Fluxes** |  |  | **(Mg ha^-1^ yr^-1^)** |  | **(Mg C ha^-1^ yr^-1^)** |  |  |
| Fine  litterfall | Leaves, small twigs (>1cm diameter), reproductive organs | 40  (25 collection dates) | 11.7 (± 0.3) | 0.42 | 4.9 (± 0.1) |  | Own data; C content (Burghouts et al. 1992) |
| Soil  respiration | Heterotrophic ecosystem respiration | 40  (7 collection dates) |  |  | 28.6 (± 1.2) |  | Own data |
| **Total stocks** |  |  | **258.3 (± 7.6)** |  | **167.9 (± 3.8)** |  |  |
