# Supplementary material for: Carbon Stocks and Fluxes in Tropical Lowland Dipterocarp Rainforests in Sabah, Malaysian Borneo
Source: PLoS One. 2012 Jan 3;7(1):e29642. doi: 10.1371/journal.pone.0029642 (PMC3250468; doi:10.1371/journal.pone.0029642)
Supplement: Table S4 — Overview of most important tree families and species in unlogged forest. (DOC) [file pone.0029642.s005.doc]

Table S4 Unlogged forest: Overview of the nine most important tree families and the ten most important species (>10 cm DBH). BA: mean (± SEM) basal area, DBH: diameter at breast height.

| **Family** | **Species** | **BA (m^2^ ha^-1^)** | **BA (%)** | **DBH range (cm)** | **Density (ha^-1^)** |
| --- | --- | --- | --- | --- | --- |
| **Dipterocarpaceae** |  | **18.24 (± 0.66)** | **61.0** | **10.1 – 170.3** | **87** |
|  | *Shorea johorensis* | 9.09 (± 0.67) | 30.4 | 10.5 – 170.3 | 21 |
|  | *Shorea parvifolia* | 6.22 (± 0.09) | 20.8 | 11.7 – 116.3 | 28 |
|  | *Parashorea malaanonan* | 1.61 (± 0.26) | 5.4 | 10.6 – 67.2 | 12 |
|  | *Hopea nervosa* | 0.99 (± 0.11) | 3.3 | 10.1 – 47.5 | 17 |
| **Meliaceae** |  | **1.91 (± 0.11)** | **6.4** | **10.0 – 32.3** | **61** |
|  | *Chisocheton sarawakensis* | 0.55 (± 0.04) | 1.8 | 10.0 – 30.0 | 17 |
|  | *Aglaia elliptica* | 0.45 (± 0.02) | 1.5 | 10.1 – 31.8 | 15 |
|  | *Aglaia macrocarpa* | 0.39 (± 0.03) | 1.3 | 11.4 – 31.0 | 10 |
| **Leguminosae** |  | **1.89 (± 0.40)** | **6.3** | **10.0 – 144.8** | **6** |
|  | *Koompassia excelsa* | 1.65 (± 0.41) | 5.6 | 144.8 | 1 |
| **Lauraceae** |  | **1.33 (± 0.09)** | **4.4** | **10.0 – 45.6** | **44** |
| **Euphorbiaceae** |  | **1.13 (± 0.08)** | **3.8** | **20.0 – 36.1** | **61** |
| **Myrtaceae** |  | **0.98 (± 0.13)** | **3.3** | **10.7 – 57.1** | **19** |
|  | *Syzygium fastigiatum* | 0.63 (± 0.12) | 2.1 | 14.1 – 57.1 | 5 |
| **Tiliaceae** |  | **0.76 (± 0.08)** | **2.5** | **10.0 – 43.1** | **21** |
|  | *Pentace laxiflora* | 0.65 (± 0.08) | 2.2 | 11.4 – 43.1 | 15 |
| **Fagaceae** |  | **0.62 (± 0.06)** | **2.1** | **11.7 – 50.5** | **10** |
| **Burseraceae** |  | **0.38 (± 0.03)** | **1.3** | **10.6 – 41.4** | **11** |
| **Others** |  | **2.67 (± 0.04)** | **8.9** | **10 – 52.0** | **99** |
| **Total** |  | **29.91 (± 0.66)** | **100** | **10 – 170.3** | **410** |
