# Supplementary material for: Carbon Stocks and Fluxes in Tropical Lowland Dipterocarp Rainforests in Sabah, Malaysian Borneo
Source: PLoS One. 2012 Jan 3;7(1):e29642. doi: 10.1371/journal.pone.0029642 (PMC3250468; doi:10.1371/journal.pone.0029642)
Supplement: Table S5 — Overview of most important tree families and species in selectively logged forest. (DOC) [file pone.0029642.s006.doc]

Table S5 Selectively logged forest: Overview of the nine most important tree families and the ten most important species (>10 cm DBH). BA: mean (± SEM) basal area, DBH: diameter at breast height.

| **Family** | **Species** | **BA (m^2^ ha^-1^)** | **BA (%)** | **DBH range (cm)** | **Density (ha^-1^)** |
| --- | --- | --- | --- | --- | --- |
| **Dipterocarpaceae** |  | **6.88 (± 0.17)** | **27.6** | **10.0 – 84.3** | **69** |
|  | *Shorea johorensis* | 1.61 (± 0.17) | 6.4 | 13.8 – 84.3 | 7 |
|  | *Shorea gibbosa* | 1.54 (± 0.14) | 6.2 | 10.6 – 72.5 | 13 |
|  | *Dryobalanops lanceolata* | 0.86 (± 0.08) | 3.4 | 12.8 – 72.1 | 7 |
|  | *Shorea fallax* | 0.67 (± 0.17) | 2.7 | 13.4 – 71.0 | 3 |
|  | *Dipterocarpus caudiferus* | 0.59 (± 0.10) | 2.4 | 9.8 – 60.5 | 10 |
| **Euphorbiaceae** |  | **5.42 (± 0.24)** | **21.7** | **10.0 – 64.0** | **107** |
|  | *Macaranga pearsonii* | 2.75 (± 0.21) | 11.0 | 17.0 – 64.0 | 24 |
|  | *Macaranga gigantea* | 1.29 (± 0.05) | 5.2 | 13.0 – 38.8 | 23 |
| **Rubiaceae** |  | **3.79 (± 0.16)** | **15.2** | **10.0 – 48.0** | **74** |
|  | *Neolamarckia cadamba* | 3.11 (± 0.13) | 12.4 | 10.2 – 48.0 | 33 |
| **Leguminosae** |  | **0.84 (± 0.10)** | **3.4** | **10.3 – 72.8** | **14** |
| **Datiscaceae** |  | **0.79 (± 0.16)** | **3.2** | **23.8 – 80.9** | **4** |
|  | *Octomeles sumatrana* | 0.79 (± 0.16) | 3.2 | 23.8 – 80.9 | 4 |
| **Lauraceae** |  | **0.75 (± 0.07)** | **3.0** | **11.1 – 59.8** | **12** |
| **Sonneratiaceae** |  | **0.71 (± 0.14)** | **2.8** | **14.2 – 77.2** | **5** |
|  | *Duabanga moluccana* | 0.71 (± 0.14) | 2.8 | 14.2 – 77.2 | 5 |
| **Sapindaceae** |  | **0.55 (± 0.06)** | **2.2** | **16.5 – 51.2** | **8** |
| **Tiliaceae** |  | **0.54 (±0.07)** | **2.2** | **11.5 – 62.1** | **8** |
| **Others** |  | **4.69 (± 0.13)** | **18.8** | **10.0 – 59.1** | **116** |
| **Total** |  | **24.96 (± 0.83)** | **100** | **10.0 – 84.3** | **417** |
