# Supplementary material for: Carbon Stocks and Fluxes in Tropical Lowland Dipterocarp Rainforests in Sabah, Malaysian Borneo
Source: PLoS One. 2012 Jan 3;7(1):e29642. doi: 10.1371/journal.pone.0029642 (PMC3250468; doi:10.1371/journal.pone.0029642)
Supplement: Table S6 — Study survey from in and around Danum Valley Conservation Area. (DOC) [file pone.0029642.s007.doc]

Table S6 Mean (±SEM) basal area, total aboveground biomass (TAGB), and aboveground carbon (TAGC) estimates from in and around Danum Valley Conservation Area.

| **History** | **Basal Area**  **(m^2^ ha^-1^)** | **TAGB**  **(Mg ha^-1^)** | **TAGC**  **(Mg C ha^-1^)** | **N** | **Study** |
| --- | --- | --- | --- | --- | --- |
| Unlogged | NA | 276 (29.0) | 138.0 (14.5) | 10 | Berry et al. 2010^a^ |
| Logged | NA | 177 (14.4) | 88.5 (7.2) | 10 | Berry et al. 2010^b^ |
| Unlogged | 35.7 (6.2) | 506.4 (60.2) | 253.2 (30.1) | 5 | Tangki & Chappell 2008^a^ |
| Logged | 18.5 (4.7) | 171.7 (67.8) | 85.9 (33.9) | 5 | Tangki & Chappell 2008^c^ |
| Unlogged | 27.5 (2.9) | 333.5 (33.2) | 166.8 (16.6) | 4 | Pinard & Putz 1996^d^ |
| Logged | NA | 147 (19.2) | 73.5 (9.6) | 4 | Pinard & Putz 1996^e^ |
| Unlogged | 29.9 (0.7) | 256 (26.8) | 128 (13.4) | 4 | Saner 2009^a^ |
| Logged | 25 (0.8) | 183.8 (5.8) | 91.9 (2.9) | 4 | Saner 2009^f^ |

^a^Unlogged forest measurements were taken at Danum Valley Conservation Area.

^b^Selective logging occurred 18 years prior to measurements.

^c^Selective logging occurred 20 years prior to measurements.

^d^Unlogged forest measurements were taken at Ulu Segama forest.

^e^Selective logging occurred 1 year prior to measurements.

^f^Selective logging occurred 22 years prior to measurements.
