# Supplementary material for: Carbon Stocks and Fluxes in Tropical Lowland Dipterocarp Rainforests in Sabah, Malaysian Borneo
Source: PLoS One. 2012 Jan 3;7(1):e29642. doi: 10.1371/journal.pone.0029642 (PMC3250468; doi:10.1371/journal.pone.0029642)
Supplement: Table S7 — Carbon content survey in soil organic matter. (DOC) [file pone.0029642.s008.doc]

Table S7 Mean (± SEM) carbon content in soil organic matter (SOM) of the Sabah Biodiversity Experiment (SBE). Samples were taken in the middle of all twelve control plots. For each soil depth layer three replicates were pooled for analysis (n=396). Carbon content was derived with the Walkley-Black method. For further details see main text.

| **Depth (cm)** | **P20** | **P24** | **P26** | **P36** | **P47** | **P57** | **P68** | **P80** | **P89** | **P95** | **P108** | **P117** | **SOM  (Mg C ha^-1^)** |
| --- | --- | --- | --- | --- | --- | --- | --- | --- | --- | --- | --- | --- | --- |
| 0–5 | 5.1 | 7.9 | 6.9 | 4.2 | 3.8 | 11.9 | 2.3 | 7.9 | 5.6 | 6.0 | 6.5 | 9.7 | **6.5 (± 0.2)** |
| 5–10 | 3.7 | 4.7 | 7.7 | 2.8 | 4.2 | 3.5 | 3.1 | 3.0 | 3.3 | 3.0 | 5.0 | 5.8 | **4.1 (± 0.1)** |
| 10–20 | 6.4 | 8.6 | 6.2 | 3.6 | 9.6 | 9.0 | 4.2 | 4.2 | 7.3 | 2.9 | 6.4 | 9.0 | **6.5 (± 0.2)** |
| 20–30 | 4.4 | 7.7 | 2.6 | 3.8 | 6.5 | 8.6 | 4.2 | 3.6 | 3.4 | 3.0 | 4.2 | 7.2 | **4.9 (± 0.2)** |
| 30–40 | 4.8 | 6.8 | 2.2 | 2.4 | 2.8 | 0.9 | 3.0 | 1.1 | 1.9 | 2.4 | 1.0 | 5.4 | **2.9 (± 0.2)** |
| 40–50 | 4.2 | 4.6 | 2.5 | 3.8 | 1.9 | 3.2 | 2.4 | 1.0 | 1.6 | 1.0 | 1.9 | 4.1 | **2.7 (± 0.1)** |
| 50–60 | 4 | 3.7 | 5.5 | 3.2 | 4.1 | 2.0 | 0.2 | 0.6 | 3.6 | 1.4 | 4.2 | 3.5 | **2.9 (± 0.2)** |
| 60–70 | 1.4 | 2.8 | 1.8 | 4.1 | 3.4 | 2.4 | 0.4 | 1.6 | 2.2 | 0.1 | 2.2 | 3.4 | **2.2 (± 0.1)** |
| 70–80 | 1.1 | 3.2 | 1.1 | 4.0 | 3.0 | 2.0 | 2.2 | 1.2 | 2.1 | 0.9 | 2.6 | 2.9 | **2.2 (± 0.1)** |
| 80–90 | 0.5 | 3.5 | 1.2 | 4.7 | 2.0 | 2.8 | 2.8 | 1.1 | 5.8 | 0.8 | 2.8 | 2.8 | **2.6 (± 0.1)** |
| 90–100 | 1.4 | 2.3 | 1.2 | 5.2 | 2.2 | 3.5 | 1.2 | 1.1 | 1.8 | 0.5 | 2.3 | 2.4 | **2.1 (± 0.1)** |
| **Total  (Mg C ha^-1^)** | **37.0** | **56.3** | **39.0** | **41.6** | **43.4** | **49.8** | **26.0** | **26.4** | **38.6** | **22.0** | **39.2** | **56.3** | **39.6 (± 0.9)** |
